# A bibliometric and visualized research on global trends of immune checkpoint inhibitors related complications in melanoma, 2011–2021

**DOI:** 10.3389/fendo.2023.1164692

**Published:** 2023-04-20

**Authors:** Hongyi Zhang, Yanlong Shi, Jianghui Ying, Yi Chen, Rong Guo, Xin Zhao, Lingling Jia, Jiachao Xiong, Fei Jiang

**Affiliations:** ^1^ Department of Plastic Surgery, Shanghai East Hospital, Tongji University School of Medicine, Shanghai, China; ^2^ Department of Breast Surgery, Yangpu Hospital, Tongji University School of Medicine, Shanghai, China; ^3^ Hepatopancreatobiliary Center, The Second Affiliated Hospital of Nanjing Medical University, Nanjing, Jiangsu, China; ^4^ Department of Biotechnology, The China-US (Henan) Hormel Cancer Institute, Zhengzhou, China; ^5^ Department of General Surgery, Fuyang Hospital of Anhui Medical University, Fuyang, China

**Keywords:** immune checkpoint inhibitor, immunotherapy, complication, melanoma, bibliometric analysis

## Abstract

**Background:**

Melanoma is a malignant tumor that originates from the canceration of melanocytes with a high rate of invasiveness and lethality. Immune escape has been regarded as an important mechanism for tumor development, while the treatment of immune checkpoint inhibitors (ICIs) is beneficial in restoring and enhancing the body’s anti-tumor immune response to kill tumor cells. To date, ICIs therapy has achieved remarkable efficacy in treating melanoma patients. Despite the significant clinical benefits of ICIs, multiple complications such as rashes, thyroiditis, and colitis occur in melanoma patients. In this study, we aim to explore the development process and trends in the field of ICIs-related complications in melanoma, analyze current hot topics, and predict future research directions.

**Methods:**

We screened the most relevant literatures on ICIs-related complications in melanoma from 2011 to 2021 in the Web of Science Core Collection (WoSCC). Using VOSviewer, CiteSpace and R language packages, we analyzed the research trends in this field.

**Results:**

A total of 1,087 articles were screened, and the USA had the highest number of publications (publications = 454, citations = 60,483), followed by Germany (publications = 155, citations = 27,743) and Italy (publications = 139, citations = 27,837). The Memorial Sloan Kettering Cancer Center had the most publications, but the Angeles Clinic and Research Institute had the highest average citation rate. Lancet oncology (IF, 2021 = 54.43) was the most prominent of all journals in terms of average citation rate. Reference and keyword cluster analysis revealed that anti-tumor efficacy, adjuvant treatment, clinical response, clinical outcome, etc. were the hotspots and trends of research in recent years.

**Conclusions:**

This study offers a comprehensive summary and analysis of global research trends on ICIs-related complications in melanoma. Over the past decade, there has been a significant increase in the number of publications on this topic. However, the safety and benefits of retreatment after the recovery of ICIs-related complications remain unknown. Therefore,the establishment of related prediction models, as well as the immunotherapy of melanoma with ICIs in combination with other adjuvant therapies, are future research hotspots.

## 1 Introduction

Melanoma is a type of cancer that arises from the malignant transformation of melanocytes, which occurs in the skin mucosa and skin pigmentation (1). Despite accounting for only 1% of skin tumors, the mortality rate of melanoma ranks first in skin tumors (2). Currently, surgery is the preferred treatment for stage I-II melanoma, with up to 80% of patients surviving for 5 years. However, for advanced and distantly metastatic malignant melanoma, the 5-year survival rate is less than 10%, making it the leading cause of melanoma-related deaths (3). Melanoma is extremely insensitive to radiotherapy, so chemotherapy has been the mainstay of treatment for surgically unresectable or advanced melanoma. However, the low therapeutic efficiency and serious toxic side effects of chemotherapeutic drugs have greatly limited their use (4, 5).

Immune checkpoints are cell surface molecules that regulate the strength and quality of the body’s immune response, including checkpoint molecules that upregulate the immune response, such as CD28 and OX-40, and inhibitory checkpoint molecules that downregulate the immune response, such as CTLA- 4 and PD-1 (6–8). Immune checkpoints play a crucial role in immune tolerance, allowing tumor cells to escape surveillance by the host immune system (9). Tumor immune escape mechanisms are extremely crucial to the development of tumorigenesis (9). Thus, the use of immune checkpoint inhibitors (ICIs) is beneficial in restoring and enhancing the body’s anti-tumor immune response to eliminate tumor cells. To date, ICIs such as the CTLA-4 inhibitor (ipilimumab), the PD-1 inhibitor (nivolumab and pembrolizumab) have been approved by the U.S. Food and Drug Administration for the treatment of metastatic melanoma and have demonstrated significant efficacy (10–12).

Despite the significant clinical benefits of ICIs, they are associated with a range of adverse effects that can affect multiple organs throughout the body (13). For example, gastrointestinal adverse reactions are more common with CTLA-4 inhibitors, and pneumonia and thyroiditis are more common with PD-1 inhibitors. Furthermore, the incidence of adverse events in immunocombination therapy is significantly higher than that in monotherapy (10, 14, 15). Numerous studies have been reported on complications related to ICIs in melanoma, but no reverent studies have analyzed the overall trend of complications.

Bibliometric analysis combines mathematics and statistics to quantitatively describe the current state of scientific research, research topics, and research trends based on unique parameters of published literature, such as country, institution, author, etc. (16). As such, this study provides a comprehensive quantitative and qualitative evaluation of studies on ICIs-related complications in melanoma over the last 10 years using bibliometrics. The aim of this analysis is to continuously explore hot topics and research trends in this field and provide directions for future research.

## 2 Materials and methods

### 2.1 Data collection

**Figure 1:**
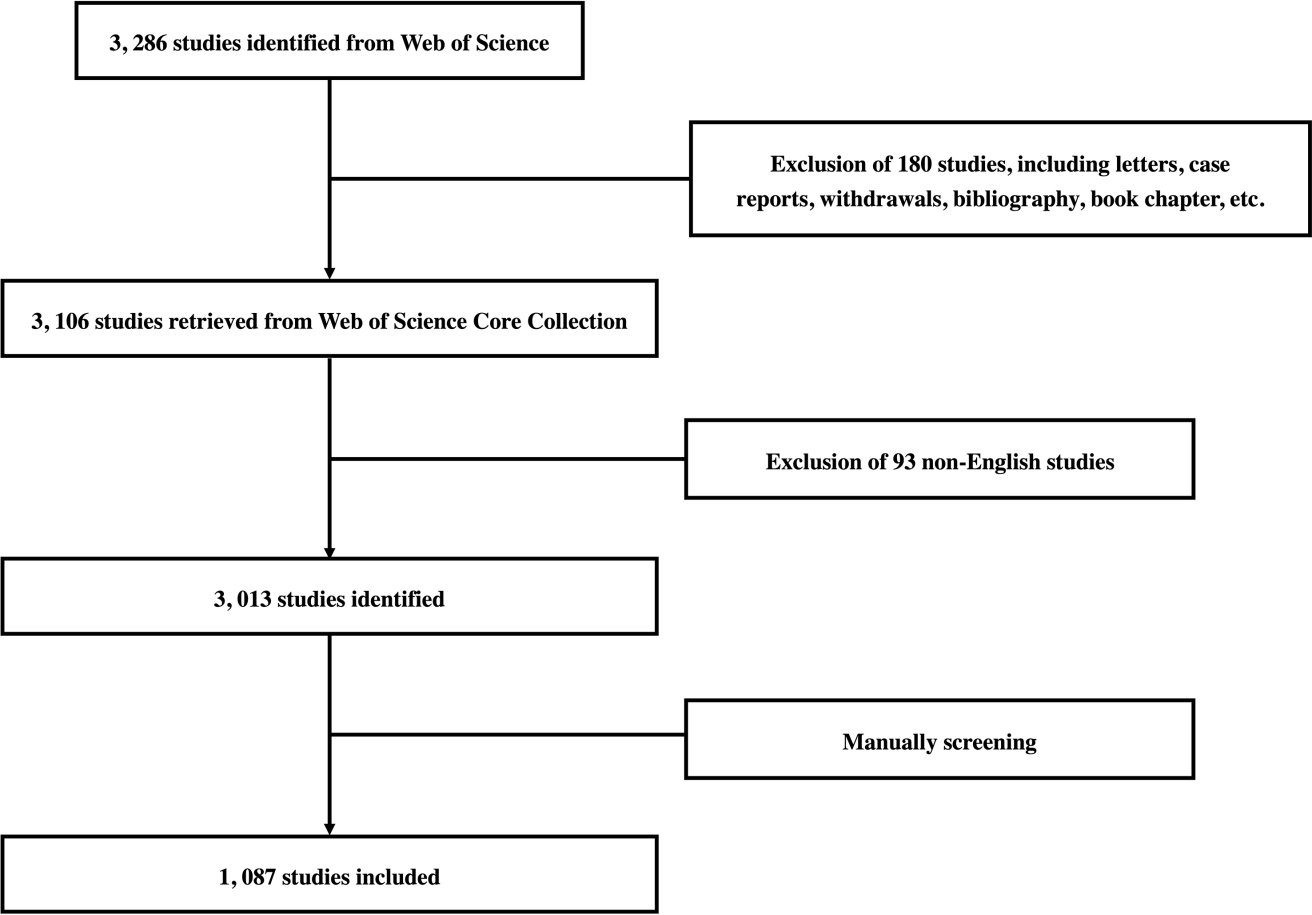
Flow chart of data collection strategies for ICIs related complications in melanoma.

The Web of Science Core Collection (WoSCC) was performed to collect data on ICIs related complications in melanoma from 2011 to 2021. Search strategies were set as follows: TS (title/abstract) = (ICI* OR immune-checkpoint inhibitor* OR immune checkpoint inhibitor* OR immune-checkpoint blockade* OR immune checkpoint blockade* OR checkpoint inhibitor* OR CPI* OR ipilimumab OR nivolumab OR pembrolizumab OR toripalimab OR lambrolizumab OR atezolizumab OR avelumab OR durvalumab) AND TS (title/abstract) = (adverse event* OR complication* OR side effect*) AND TS (title/abstract) = (melanoma OR chronic melanoma OR metastatic melanoma OR malignant melanoma OR advanced melanoma) AND Publication Date = (2011-01-01 to 2021-12-31) AND Language = (English). Subsequently, the studies most relevant to ICIs related complications in melanoma were manually screened. The detail of the search process and included studies was shown in **Figure 1** and **Supplementary Material S1**. All literature searches were conducted within one day (03 July 2022) to avoid bias related to database updates. The types of original article and review publication were screened to include them. The first and second authors independently retrieved data and all data, including the number of publications and citations, titles, countries/regions, affiliations, etc., were downloaded from WoSCC for further analyses.

### 2.2 Bibliometric analysis

The bibliometric analysis was performed by using Microsoft Excel 2021, VOSviewer (version 1.6.17), CiteSpace V (version 6.1.2) and R language package from the online tool website (http://www.bioinformatics.com.cn/srplot). Based on the data downloaded from the WoSCC, the annual of publications and citation was performed by Microsoft Excel 2021; The bibliometric network or density map of intercountry and organisation cooperation, linkage of journal, and cooccurrence of keywords was visualised by VOSviewer. In term of the county/region and journal analysis, the minimum threshold of inclusion criteria was set as 5, 33 countries and 49 journals were included and visualized, respectively. In term of organization, author and keyword analysis, the minimum threshold of inclusion criteria was set as 10 with 83 organizations, 69 authors and 162 keywords included and visualized, respectively; The cooperation among authors, cluster analysis and bursts of references and keywords were employed by CiteSpace V; The geographical distribution of publications worldwide, the radar charts of the top 10 productive institutions, and journals were used by using R language packages. The latest impact factors (IF) and zoning were obtained from the newest edition of the Journal Citation Report (JCR, 2021).

## 3 Results

### 3.1 Overall distribution and global contributions

**Figure 2:**
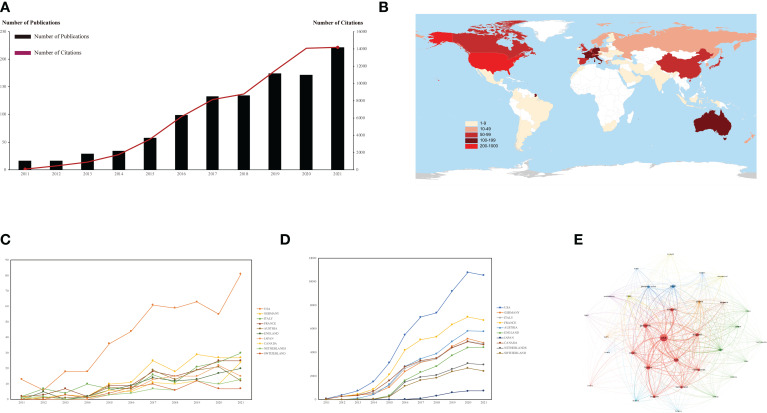
Publications distribution about ICIs related complications in melanoma worldwide. **(A)** Global annual publication and citation status of ICIs related complications in melanoma. **(B)** Geographical distribution of global publications. **(C)** Annual publication trends in top 10 country/region with the most publications. **(D)** Annual citation trends in top 10 country/region with the most publications. **(E)** The cluster network visualization of the country/region by VOSviewer.

A total of 890 original articles and 197 reviews associated with complications related complications in melanoma were selected. The annual trend of the publication and citation has been increasing steadily since 2011 (publications = 16, citations = 98), with citations peaking in 2021 (publications = 221, citations = 14, 182), although publications are slightly decrease in 2018 (publications = 135, citations = 8, 775) and 2020 (publications = 172, citations = 14, 060) (**Figure 2A**). The rapid growth in the number of publications and citations means that more researchers are investing and paying attention to this field.

Based on the publishing number, as shown in the country/region distribution map (**Figure 2B**) and top 10 most productive countries/regions were listed in **Table 1**, the top 3 countries were USA (n = 454, 41.77%), Germany (n = 155, 14.26%) and Italy (n = 139, 12.79%), respectively. The annual trend for publications and citations in the United States from 2012 to 2021 has been steadily increasing and consistently well above other countries (**Figures 2C, D**). At the peak of publications in 2021, the number of publications in the USA (publications = 81) was 2.7 times that of the second-placed Italy (publications = 30); at the peak of citations in 2020, the number of citations in the USA (citations = 10, 559) was 2.09 times that of the second-placed Germany (citations = 5, 161). The annual trend of publications in top 10 most productive countries except the USA is generally upward, but there are small fluctuations from year to year. The annual trend of citations for the top 10 most productive countries, all with significant year-over-year increases through 2021. Furthermore, an extensive network of cooperation between countries is shown in **Figure 2E**. The United States is located at the central node of the cooperation network and has close cooperation with many countries such as England, France, Canada, and Australia. It can be seen that the USA is the leader in the field of ICIs-related complications in melanoma, with a much higher number of publications and citations than any other country.

**Table 1:** Contributed publications in the top 10 countries.

Country	Publications	Citations	Average citation rate	% of (1087)
USA	454	60,483	133.22	41.77%
GERMANY	155	27,743	178.99	14.26%
ITALY	139	27,837	200.27	12.79%
FRANCE	134	41,140	307.01	12.33%
AUSTRIA	103	30,831	299.33	9.48%
ENGLAND	98	21,584	220.24	9.02%
JAPAN	89	2,813	31.61	8.19%
CANADA	83	28,402	342.19	7.64%
NETHERLANDS	59	15,506	262.81	5.43%
SWITZERLAND	58	13,434	231.62	5.34%

### 3.2 Analysis of institutions and authors

**Figure 3:**
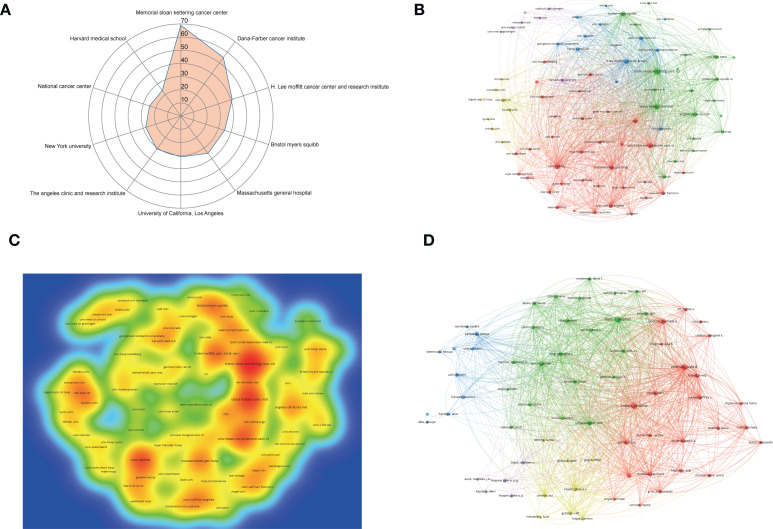
Visualization analysis of institutions and authors. **(A)** Radar map of the top 10 productive institutions. **(B)** The cluster network visualization of cooperation among institutions. **(C)** Density map of cooperation among institutions. **(D)** The cluster network visualization of authors.

A total of 1, 919 institutions have conducted studies on ICIs-related complications in melanoma. The top 10 institutions with the most publications were listed in **Table 2**. Memorial Sloan Kettering Cancer Center (n = 69) is the most productive institution, followed by Dana-Farber Cancer Institute (n = 55) and H. Lee Moffitt Cancer Center and Research Institute (n = 41) (**Figure 3A**). VOSviewer was then used to analyze and visualize the extensive network and density map of cooperation between institutions (**Figures 3B, C**). Memorial Sloan Kettering Cancer Center, Dana-Farber Cancer Institute, University of Texas MD Anderson Cancer Center and Sydney University were the hottest publication centers with the strongest ties to other institutions.

**Table 2:** Top 10 institutions with the most publications.

Institutions	Country	Publications	Citations	Average citation rate
Memorial sloan kettering cancer center	USA	69	22,307	323.29
Dana-Farber cancer institute	USA	55	14,368	261.24
H. Lee moffitt cancer center and research institute	USA	41	4,265	104.02
Bristol myers squibb	USA	35	10,476	299.31
Massachusetts general hospital	USA	35	4,551	130.03
University of California, Los Angeles	USA	31	10,952	353.29
The angeles clinic and research institute	USA	31	16,730	539.68
New York university	USA	28	4,333	154.75
National cancer center	Korea	25	646	25.84
Harvard medical school	USA	24	590	24.58

A total of 6, 140 authors have published articles on ICIs-related complications in melanoma, with the top 10 authors listed in **Table 3**. Hodi, F. Stephen was the most productive author with 41 publications, followed by Wolchok, Jedd D. with 40 publications, and Robert, Caroline with 35 publications. Based on the number of citations, Wolchok, Jedd D. was the most influential author with 17, 191 citations, followed by Robert, Caroline with 15, 984 citations and Postow, Michael A. with 10, 104 citations (publications = 29). The extensive network of cooperation between authors showed that the number of author publications determines, to a certain extent, the closeness of cooperation between authors (**Figure 3D**).

**Table 3:** Top 10 authors with the most publications.

Author	Document	Citations	Average citation rate
Hodi, F. Stephen	41	9,075	221.34
Wolchok, Jedd D.	40	17,191	429.78
Robert, Caroline	35	15,984	456.69
Postow, Michael A.	29	10,104	348.41
Larkin, James	26	6,811	261.96
Schadendorf, Dirk	25	3,772	150.88
Long, Georgina V.	18	7,067	392.61
Weber, Jeffrey S.	20	5,258	262.90
Hassel, Jessica C.	21	1,853	88.24
Garbe, Claus	20	4,703	235.15

### 3.3 Analysis of journals

**Figure 4:**
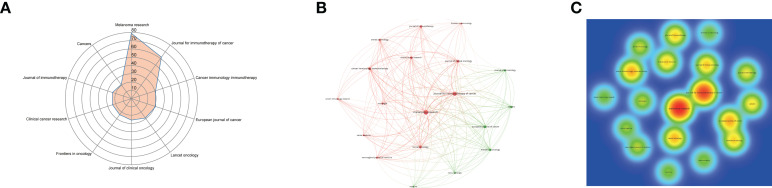
Visualization analysis of journals. **(A)** Radar map of the top 10 productive journals. **(B)** The cluster network visualization of journals. **(C)** Density map of journals.

A total of 325 journals accepted and published articles on ICIs-related complications in melanoma, with the top 10 journals listed in **Table 4**. Melanoma research (IF = 3.20) was the most productive journal with 79 publications, followed by Journal for Immunotherapy of Cancer (IF = 12.47) with 62 publications and Cancer Immunology Immunotherapy (IF = 6.63) with 31 publications (**Figure 4A**). The extensive network and density map of cooperation between journals were analyzed (**Figures 4B, C**), which showed that Melanoma Research and Journal for Immunotherapy of Cancer were the most thermal publication centers with the strongest ties to other journals.

**Table 4:** Top 10 journals with the most publications.

Journal	Document	Citations	Average citation rate	IF (2021)
Melanoma research	79	1,298	16.43	3.20
Journal for immunotherapy of cancer	62	1,546	24.94	12.47
Cancer immunology immunotherapy	31	1,234	39.81	6.63
European journal of cancer	31	882	28.45	10.00
Lancet oncology	29	10,486	361.59	54.43
Journal of clinical oncology	26	4,259	163.81	50.72
Frontiers in oncology	24	192	8.00	5.74
Clinical cancer research	24	2,138	89.08	13.80
Journal of immunotherapy	24	482	20.08	4.91
Cancers	21	157	7.48	6.58

### 3.4 Analysis of cited and co-cited references

Citation and co-citation analysis of the literature can help understand the most influential research in the field and guide the basic direction of future research. The top 10 publications with the most citations are listed in **Table 5**, and the top 10 publications with the most co-citations are presented in **Table 6**. The article with most citation (n = 5, 104) and co-citation (n = 293) was published by Larkin J et al. (14) in 2015 who revealed that compared to ipilimumab, Nivolumab alone or in combination with ipilimumab in patients with previously untreated metastatic melanoma significantly improved progression-free survival by enabling complementary activity between PD-1 and CTLA-4 blockade. Followed by Robert C et al. (17) in 2015 (citation = 3, 602, co-citation = 267) who revealed that pembrolizumab significantly prolongs progression-free survival and overall survival in patients with advanced melanoma and has less high-grade toxicity than ipilimumab.

**Table 5:** Top 10 publications with the most citations.

Title	First author	Journal	IF/JCR zoning	Publication year	Total citations
Combined Nivolumab and Ipilimumab or Monotherapy in Untreated Melanoma	Larkin J	NEW ENGLAND JOURNAL OF MEDICINE	176.08/Q1	2015	5,104
Nivolumab in Previously Untreated Melanoma without BRAF Mutation	Robert C	NEW ENGLAND JOURNAL OF MEDICINE	176.08/Q1	2015	3,656
Pembrolizumab versus Ipilimumab in Advanced Melanoma	Robert C	NEW ENGLAND JOURNAL OF MEDICINE	176.08/Q1	2015	3,602
Ipilimumab plus Dacarbazine for Previously Untreated Metastatic Melanoma	Robert C	NEW ENGLAND JOURNAL OF MEDICINE	176.08/Q1	2015	3,158
Nivolumab plus Ipilimumab in Advanced Melanoma	Wolchok JD	NEW ENGLAND JOURNAL OF MEDICINE	176.08/Q1	2015	2,990
Safety and Tumor Responses with Lambrolizumab (Anti-PD-1) in Melanoma	Hamid O	NEW ENGLAND JOURNAL OF MEDICINE	176.08/Q1	2015	2,525
Overall Survival with Combined Nivolumab and Ipilimumab in Advanced Melanoma	Wolchok JD	NEW ENGLAND JOURNAL OF MEDICINE	176.08/Q1	2015	1,926
Nivolumab and Ipilimumab versus Ipilimumab in Untreated Melanoma	Postow MA	NEW ENGLAND JOURNAL OF MEDICINE	176.08/Q1	2015	1,894
Nivolumab versus chemotherapy in patients with advanced melanoma who progressed after anti-CTLA-4 treatment (CheckMate 037): a randomised, controlled, open-label, phase 3 trial	Weber JS	LANCET ONCOLOGY	54.43/Q1	2015	1,814
Anti-programmed-death-receptor-1 treatment with pembrolizumab in ipilimumab-refractory advanced melanoma: a randomised dose-comparison cohort of a phase 1 trial	Robert C	LANCET	202.73/Q1	2015	1,299

**Table 6:** Top 10 publications with the most co-citations.

Title	First author	Journal	IF/JCR zoning	Publication year	Co-citations number
Combined Nivolumab and Ipilimumab or Monotherapy in Untreated Melanoma	Larkin J	NEW ENGLAND JOURNAL OF MEDICINE	176.08/Q1	2015	293
Pembrolizumab versus Ipilimumab in Advanced Melanoma	Wolchok JD	NEW ENGLAND JOURNAL OF MEDICINE	176.08/Q1	2015	267
Nivolumab in previously untreated melanoma without BRAF mutation	Robert C	NEW ENGLAND JOURNAL OF MEDICINE	176.08/Q1	2015	234
Overall Survival with Combined Nivolumab and Ipilimumab in Advanced Melanoma	Wolchok JD	NEW ENGLAND JOURNAL OF MEDICINE	176.08/Q1	2017	150
Nivolumab versus chemotherapy in patients with advanced melanoma who progressed after anti-CTLA-4 treatment (CheckMate 037): a randomised, controlled, open-label, phase 3 trial	Weber JS	LANCET ONCOLOGY	54.43/Q1	2015	144
Ipilimumab plus dacarbazine for previously untreated metastatic melanoma	Robert C	NEW ENGLAND JOURNAL OF MEDICINE	176.08/Q1	2011	130
Improved survival with ipilimumab in patients with metastatic melanoma	Hodi FS	NEW ENGLAND JOURNAL OF MEDICINE	176.08/Q1	2010	130
Nivolumab and ipilimumab versus ipilimumab in untreated melanoma	Postow MA	NEW ENGLAND JOURNAL OF MEDICINE	176.08/Q1	2015	117
Pembrolizumab versus investigator-choice chemotherapy for ipilimumab-refractory melanoma (KEYNOTE-002): a randomised, controlled, phase 2 trial	Ribas A	LANCET ONCOLOGY	54.43/Q1	2015	108
Adjuvant Nivolumab versus Ipilimumab in Resected Stage III or IV Melanoma	Weber J	NEW ENGLAND JOURNAL OF MEDICINE	176.08/Q1	2017	100

**Figure 5:**
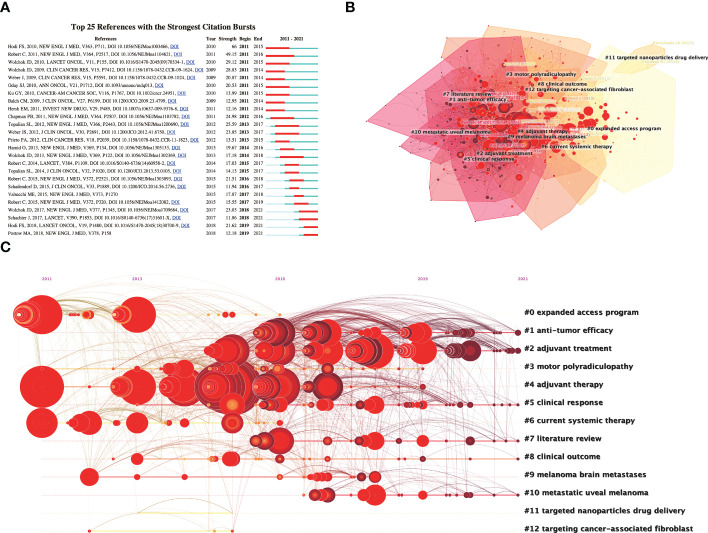
Visualization analysis of cited and co-cited references. **(A)** The top 25 co-cited references with the strongest citation bursts. **(B)** Cluster analysis of co-cited references. **(C)** Timeline visualization of co-cited references cluster analysis result from 2011 to 2021.

Based on the cocitation network, a reference burst analysis was performed, and the top 25 cocited references with the strongest citation bursts are shown in **Figure 5A**. The article with highest bursts strength (49.15) was published by Robert C et al. (18) in 2011 who revealed that the combination of ipilimumab with dacarbazine significantly improved overall survival in patients with previously untreated metastatic melanoma, but increased the incidence of hepatic impairment. Then, a title cluster analysis was performed to summarize the references in the co-citation network to understand the frontier directions. Thirteen different clusters were divided from the cocitation network of references, including the expanded access programme, antitumour efficacy, adjuvant treatment, motor polyradiculopathy, adjuvant therapy, clinical response, current systemic therapy, review of the literature, clinical outcome, brain metastases of melanoma, metastatic uveal melanoma, targeted nanoparticles and targeting cancer-associated fibroblast (**Figure 5B**). According to the clustering display of the timeline (**Figure 5C**), anti-tumor efficacy, adjuvant treatment, clinical response, literature review, clinical outcome and metastatic uveal melanoma were recent research topics of strong concerned to researchers.

### 3.5 Analysis of keywords

**Figure 6:**
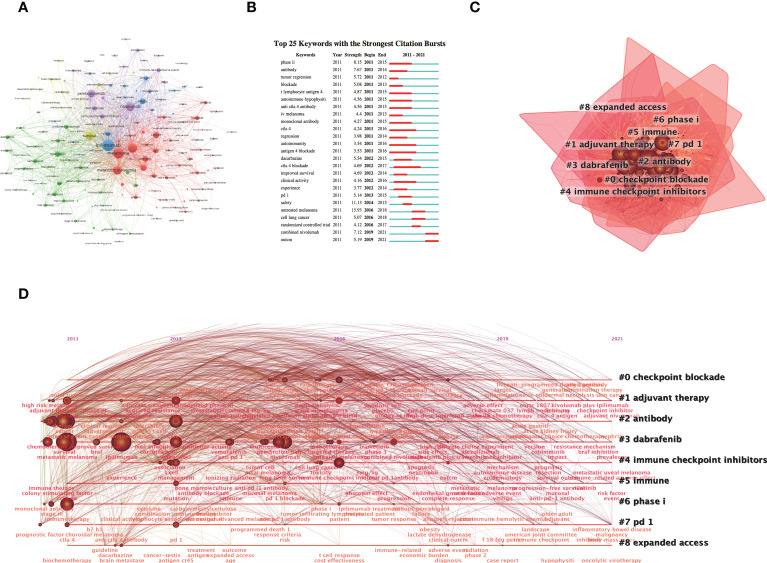
Visualization analysis of keywords. **(A)** The cluster network visualization of keywords. **(B)** The top 25 keywords with the strongest citation bursts. **(C)** Cluster analysis of keywords. **(D)** Timeline visualization of keywords cluster analysis result from 2011 to 2021.

Keywords are a generalization of the central idea of the research, and keyword co-occurrence analysis is helpful to understand the internal relationship between keywords in the field of ICIs related complications in melanoma. The extensive network of co-occurrence keywords was visualized by VOSviewer (**Figure 6A**). Ipilimumab, metastatic melanoma, survival, nivolumab, pembrolizumab and adverse event were the most co-occurrence keywords with the strongest ties to other keywords. Based on the keyword co-occurrence network, a keyword burst analysis was performed, and the top 25 keyword co-occurrence with the strongest citation bursts are shown in **Figure 6B**. It showed that the keywords with the highest burst strength were untreated melanoma (15.93), followed by safety (11.13) and phase II (8.15). Then, a cluster analysis of keywords was performed, and nine different clusters were divided from the co-occurrence network of keywords, including checkpoint blockade, adjuvant therapy, antibody, dabrafenib, immune checkpoint inhibitors, phase I, pd-1 and expanded access (**Figure 6C**). According to the timeline clustering display (**Figure 6D**), checkpoint blockade, adjuvant therapy, antibody, dabrafenib, pd-1, and expanded access were the key points of research that researchers have been paying close attention to since 2011.

## 4 Discussion

Melanoma ranks first in invasiveness and lethal type of skin tumors, and its incidence is increasing (19). While over 95% of early-stage melanomas go into remission with surgical treatment, but the prognosis for advanced and metastatic melanoma is extremely poor, with a median survival of 6-9 months and an overall survival rate of less than 10% at 5 years (2). Therefore, the development of drugs for the treatment of progressive or advanced melanoma has always been a hot research topic.

Immune escape has been regarded as an important mechanism for tumor development (9). Tumor cells suppress T-cell immune function for immune escape by activating immune checkpoints and blocking the antigen-presenting function in the tumor immune process (20). Tumor immunotherapy is a treatment method that recognizes and kills tumor cells by mobilizing the immune system to activate adaptive or innate immunity (21–23). Among these methods, ICIs therapy has achieved remarkable efficacy in the treatment of melanoma patients (24). Despite its significant clinical benefits of ICIs, multiple complications such as rashes, thyroiditis, and colitis occur in melanoma patients (25). To date, numerous studies have been reported on ICIs-related complications in melanoma, but no reverent studies have analyzed the overall trend of complications. In order to explore the development process and trends in the field of ICIs-related complications in melanoma, analyze current hot topics and predict future research directions, a bibliometric analysis of relevant literatures from 2011 to 2021 was conducted.

A bibliometric analysis of global publications and citations in the field of ICIs related complications in melanoma over the past 10 years found that the number of articles and citations in the last decade showed an overall increasing trend, although the number of papers published in 2020 has declined slightly compared with 2019 (**Figure 2A**). Interestingly, to exclude the interference of publication volume on being cited, we calculated the annual of average citation rate, which was much higher in 2020 (81.74) than in 2019 (66.01) and 2021 (64.17). Then, the annual and overall trend of publications and citations in top 10 most productive countries were analyzed and showed that the USA is significantly higher than other countries in all aspects. This result is consistent with the institution analysis, where 90% of the top 10 most productive institutions are located in the USA (**Table 2**). In particular, although Canada has only 83 publications, its average citation rate (342.19) is much higher than that of the USA (133.22), followed by France (307.01). It is evident that although the USA is a leader in the field of ICIs-related complications in melanoma, it still needs to pay attention to improving the overall quality of its publications.

In terms of author distribution (**Table 3**), Hodi, F. Stephen (Dana-Farber cancer institute) from USA is the most productive and has a certain number of citations. Wolchok, Jedd D. (Memorial Sloan Kettering Cancer Center) from USA ranked second in the number of published papers, but has the most citations among the top publishing authors. Robert, Caroline (Institut Gustave Roussy) from France ranked third in the number of publications and citations, but has the most average citation rate among the top publishing authors. Robert, Caroline team found that nivolumab significantly improved overall and progression-free survival compared to dacarbazine in previously untreated patients with metastatic melanoma without BRAF mutations (26). However, the study found that the combination of nivolumab and ipilimumab increased the incidence of grade 3-4 adverse events, with colitis, diarrhea, and elevated alanine aminotransferase being the most common (26). This result is consistent with the findings of Larkin, James team (14). In the same year, Robert, Caroline team found that pembrolizumab significantly prolongs progression-free survival and overall survival in patients with advanced melanoma and has less high-grade toxicity than ipilimumab (17). Among them, the most common immune-related adverse events with pembrolizumab were hypothyroidism and hyperthyroidism, and colitis and hypophysitis with ipilimumab (17). It is evident that the efficacy of combination therapy with ICIs is superior to that of monotherapy, but with a significantly higher incidence of associated adverse events.

Based on the references burst analysis and keywords burst analysis, the highest bursts strength in recent years (23.03) article was published by Wolchok JD et al. (27) in 2017 who revealed that the combination of nivolumab with ipilimumab or with nivolumab alone significantly improved overall survival compared with ipilimumab alone in patients with advanced melanoma. However, the study found that the combination of nivolumab and ipilimumab increased the incidence of adverse events, with select adverse events being the most common with skin-related adverse events; grade 3-4 adverse events were most common with gastrointestinal adverse events (27). Similarly, combined nivolumab is the keyword with the highest burst strength (7.12) that has appeared in recent years. A title cluster analysis of the references and a keyword co-occurrence cluster analysis of the publications were performed to analyze the current hot topics of ICIs related complications in melanoma. For the grouping analysis of references on the timeline (**Figure 5C**), researchers seemed to focus more on antitumour efficacy, adjuvant treatment, clinical response, literature review, clinical outcome, and metastatic uveal melanoma. For instance, identification of biomarkers in patients benefiting from ICIs is a current research priority, and adverse events associated with ICIs are considered as a potential clinical biomarker. Das S et al. (28) found a correlation between immune-related adverse events and antitumor efficacy of immune checkpoint inhibitors, with patients experiencing adverse events showing significant improvements in progression-free survival, overall survival, and overall response rate. For the cluster analysis of keywords on the timeline (**Figure 6D**), it is not difficult to find that ICIs in combination with other adjuvant therapies for the immunotherapy of melanoma are a current research hotspot.

This study identified the relevant publications on ICIs related complications in melanoma in the WoSCC database in the past 10 years, and comprehensively analyzes the current hotspot trend. However, this study still has some limitations. For example, we only included publications in English, which will result in the exclusion of many non-English quality publications. Therefore, a multicentre collaboration with researchers from other countries could be followed up to conduct a broader and more in-depth study.

## 5 Conclusion

In summary, this study offers a thorough quantitative and qualitative evaluation of studies about complications related to ICIs in melanoma from 2011 to 2021. Over the past decade, there has been a substantial increase in the number of publications on this topic. ICIs-related complications can be used as clinical markers for the anti-tumor efficacy of ICIs, thus the establishment of related prediction models and the immunotherapy of melanoma with ICIs in combination with other adjuvant therapies are the future research hotspots.

## Data availability statement

The original contributions presented in the study are included in the article/**Supplementary Material**. Further inquiries can be directed to the corresponding authors.

## Author contributions

JX and FJ designed the study. LJ and JY performed and drafted the experiment. HZ, RG, XZ, YS, and YC revised the manuscript, and all authors approved the final version of the manuscript. All authors contributed to the article and approved the submitted version.
